# Integrated biotechnological and artificial intelligence innovations for plant improvement

**DOI:** 10.3389/fpls.2025.1736707

**Published:** 2025-12-11

**Authors:** Haibo Wu, Man Luo, Yubin Liu, Jinghao Yang, Yunpeng Cao

**Affiliations:** 1 School of Health and Nursing, Wuchang University of Technology, Wuhan, China; 2 Guangxi Colleges and Universities Key Laboratory for Cultivation and Utilization of Subtropical Forest Plantation, Guangxi Key Laboratory of Forest Ecology and Conservation, College of Forestry, Guangxi University, Nanning, China; 3 Key Laboratory of National Forestry and Grassland Administration on Cultivation of Fast-Growing Timber in Central South China, State Key Laboratory for Conservation and Utilization of Subtropical Agro-Bioresources, College of Forestry, Guangxi University, Nanning, China

**Keywords:** artificial intelligence, plant improvement, biotechnological, AI-driven, AI-powered design

## 1 Introduction

Facing climate change and a growing population, Artificial Intelligence (AI) is leading a revolution in plant breeding, offering unprecedented opportunities to secure our future food supply. The global food system faces dual challenges: a rising population projected to reach 10 billion by 2050 is increasing demand for food, feed, and fiber (Van Dijk et al., 2021), while climate change simultaneously erodes limited arable land and threatens food system stability through extreme weather, soil salinization, and evolving pests and diseases (Hasegawa et al., 2021; Singh et al., 2023). Accelerating plant breeding to develop higher-yielding, better-quality, and more resilient crop varieties is a strategic imperative for global food security and sustainable development. Over the past century, plant breeding has progressed through several technology-driven leaps that have greatly increased agricultural productivity. These advances include early 20th-century hybrid breeding that leveraged heterosis in maize (Crow, 1998), the semi-dwarf varieties of the 1960s “Green Revolution” (Pingali, 2012), and the subsequent genetic engineering of insect-resistant and herbicide-tolerant crops (Lu et al., 2012; Li et al., 2025). However, these once-revolutionary methods are proving too slow for today’s complex challenges. Traditional breeding is inefficient for improving multi-gene traits like yield and stress resistance, and even with accelerated techniques like marker-assisted selection, bringing a superior gene from discovery to the field can take over a decade.

To overcome the bottlenecks of speed and accuracy in plant breeding, the integration of modern biotechnology and AI is driving a technological revolution to reshape the entire process in response to rapid environmental changes. Transcending the limitations of natural variation and random mutagenesis, genome editing technologies like CRISPR-Cas provide the surgical precision to rewrite the genome, enabling the rapid “*de novo* domestication” of wild plants and vastly expanding the available genetic pool (Li et al., 2018). Simultaneously, AI, particularly deep learning, is deciphering life’s complex code. It can mine massive multi-omics data for “elite alleles,” predict protein structures with models like AlphaFold (Abramson et al., 2024), and even design novel functional proteins (Kortemme, 2024). This is transforming plant science from a discipline of observation and experimentation into a precise science guided by prediction and design.

Overall, by combining AI’s predictive design with biotechnology’s precise implementation, a “Design-Build-Test-Learn” (DBTL) closed-loop accelerator is formed. In this cycle, AI models analyze vast genetic, phenotypic, and environmental data to propose optimal designs. Biotechnology tools like gene editing then build these designs into organisms. High-throughput phenotyping with drones and sensors rapidly tests the results, feeding new data back to the AI for continuous learning and optimization. This self-improving process promises to shorten breeding cycles from years to months and enable the improvement of previously unattainable complex traits. This article will highlight how AI, in synergy with gene editing and high-throughput phenotyping, is creating a ‘DBTL’ cycle that accelerates breeding cycles and enables the improvement of complex traits, while also exploring the associated challenges and opportunities.

## 2 AI-driven precision genomics and allele mining

Advances in sequencing technology have generated unprecedented amounts of multi-omics data, spanning genomics, transcriptomics, and proteomics. For example, researchers have sequenced the genomes of over ten species and accumulated vast transcriptome data from diverse tissues and developmental stages in the Rosaceae family (Cao et al., 2024, Cao et al., 2025; Jiang et al., 2025). However, the real challenge lies in identifying the key genes and genetic variations related to target traits from high-dimensional and complex data, since data itself is not knowledge. AI offers a transformative solution for complex genomic analysis. Recent studies demonstrate that advanced AI models, particularly those employing deep learning, can efficiently integrate large-scale multi-omics data to achieve outcomes that surpass the capabilities of traditional bioinformatics, such as predicting gene function, identifying crucial regulatory elements in non-coding regions, and deciphering intricate gene regulatory networks (Li et al., 2025). For example, Wu et al. developed the AutoGP AI-powered breeding platform by integrating multi-omics data from maize (genomics, transcriptomics, and metabolomics) to guide the selection of superior hybrid varieties and enhance breeding efficiency (Wu et al., 2025).

AI identifies subtle epistatic genetic combinations overlooked by traditional breeding, driving the shift from marker-assisted selection to predictive breeding. AI empowers us to simulate and predict the phenotypic performance of new plant varieties in specific environments within a computer before they are even created. This offers a significant advantage in plant breeding, drastically shortening breeding cycles and improving success rates (Li et al., 2025). However, a clear weakness lies in the fact that the performance of AI models heavily relies on large-scale, high-quality, and precisely annotated training datasets. For many orphan crops or traits that are not well-studied, the scarcity of effective data is a major bottleneck limiting the application of AI (Li et al., 2025).

## 3 Synergies of gene editing and AI-powered design

The advent of CRISPR-Cas technology has enabled precise, “surgical scalpel” level modifications to plant genomes (Kim et al., 2025; Li et al., 2025; Ruffolo et al., 2025). Now, AI is equipping this “scalpel” with a smart navigation system. From designing efficient guide RNAs (gRNAs) with low off-target rates to predicting potential genome-wide off-target effects of gene editing, AI tools are significantly improving the efficiency and safety of gene editing (Ruffolo et al., 2025). For example, by constructing a curated dataset of CRISPR operons, comprising Cas proteins, CRISPR arrays, trans-activating CRISPR RNA (tracrRNA), and Protospacer Adjacent Motifs (PAMs), Ruffolo et al. (2025) engineered novel proteins with diversity far exceeding natural variation and predicted structural effectiveness. In addition, AI has also begun to venture into the field of *de novo* design. By learning from vast amounts of protein structure and function data, AI models can design proteins with entirely new functions that do not exist in nature (Kortemme, 2024). For example, GENERA, a *de novo* design algorithm by Lamanna et al. (2023), integrates deep learning with a genetic algorithm to rapidly generate focused compound libraries that score higher than known ACE-2 binders (Lamanna et al., 2023). In plant improvement, this means we can design Rubisco enzymes with higher photosynthetic efficiency, metabolic enzymes capable of degrading novel herbicides, or disease-resistant proteins with broad-spectrum resistance to specific pathogens. The synergy of AI and gene editing is transforming plant breeding from modification to creation, enabling the programming of novel biological functions to develop breakthrough crop traits beyond the limitations of natural variation (Li et al., 2025). However, challenges remain: efficient and universally applicable gene editing delivery systems are still technically challenging in many plants. The scarcity of high-quality training data, especially when the data types are diverse, the standardization level is low, and biases exist, limits the predictive power of AI models. Furthermore, the lack of globally harmonized regulations surrounding gene-edited crops creates uncertainty for their practical application due to complex regulatory landscapes (Li et al., 2025).

## 4 High-throughput phenotyping and predictive phenomics

The phenotype, as the ultimate manifestation of the interaction between genes and the environment, serves as the direct basis for selection by breeders (Li et al., 2025). However, the labor-intensive and subjective nature of traditional phenotypic assessment methods constitutes a major bottleneck in modern breeding, impeding the efficient and accurate selection of desirable traits. High-throughput phenotyping platforms, composed of drones, ground robots, multispectral cameras, and various sensors, capture plant dynamic data throughout the growing season with unprecedented scale and precision. AI, particularly computer vision, is crucial for unlocking the value of this massive phenotyping data. AI algorithms can automatically and accurately measure thousands of phenotypic traits, such as plant height, leaf area index, disease spot area, fruit count, and color. More importantly, by integrating continuous phenotyping data, environmental data, and genomic data, AI can construct dynamic genotype-phenotype (G2P) prediction models (Danilevicz et al., 2022; Sharma and Goel, 2025). The integration of high-throughput phenotyping and AI analysis is paving the way to bridge the “last mile” between genotype and phenotype (Sheikh et al., 2024). Breeders can leverage these models to predict the field performance of plants with specific genotypes across different years and regions, enabling more informed breeding decisions. The advantage lies in transforming phenotype identification from an “art” into a precise “science” (Sheikh et al., 2024). However, the challenges should not be underestimated: the initial investment in establishing high-throughput phenotyping platforms is substantial. Furthermore, integrating data from diverse sensors and spatiotemporal scales, while eliminating environmental noise, presents a significant computational challenge.

## 5 Discussion

The convergence of biotechnology and artificial intelligence is more than just a linear combination of technologies; it’s creating a revolutionary “Design-Build-Test-Learn” closed-loop accelerator (Alemu et al., 2024). Data from each breeding cycle, from AI-driven gene discovery to high-throughput phenotyping, continuously refines the AI model. By continuously learning, AI improves its predictive accuracy to drive more efficient, forward-thinking designs. This shift from passive adaptation to active creation holds potential far surpassing any previous agricultural revolution (Li et al., 2025). Li et al. (2025) envision an “AI-assisted crop design” model serving as a “smart co-pilot” for future breeders by integrating vast multi-modal data to understand and predict the genetic basis of complex traits. More importantly, it can optimize the search across millions of potential genetic combinations based on breeding goals to output a comprehensive improvement strategy, detailing everything from specific gene editing targets to blueprints for novel protein designs (Wei et al., 2021; Li et al., 2025; Wu and Xie, 2025). This capability enables the systematic use of minor-effect alleles and breaks linkage drag, overcoming challenges that are difficult for traditional breeding methods to address. However, realizing this vision requires overcoming key bottlenecks, the most critical being data. The performance of AI models depends on the scale, quality, and diversity of training data, yet high-quality, standardized multi-omics and phenomics datasets are scarce, especially for “orphan crops” vital to regional food security (Wu and Xie, 2025; Yetgin, 2025). A global plant science data sharing consortium is therefore essential, demanding not only technological breakthroughs but also open collaboration between research institutions, governments, and businesses (Gibbs et al., 2019). Without this data foundation, even the most advanced algorithms are ineffective. Secondly, we must navigate complex ethical, regulatory, and social challenges. On this front, as Li et al. (2025) noted, regulatory policies on gene-edited crops are becoming more scientific and rational in many countries, creating favorable conditions for new technologies. In addition, cutting-edge technologies like *de novo* designed proteins require rigorous biosafety and ecological impact assessments, necessitating urgent regulatory frameworks (Wu et al., 2023; Kortemme, 2024; Li et al., 2025). To build public trust, the scientific community must transparently communicate the science, potential benefits, such as reduced pesticide use and enhanced nutrition, and risk management measures to the public and policymakers. To bridge the widening digital divide, international cooperation, open-source tools, and knowledge sharing are essential to ensure these advanced technologies benefit everyone (Li et al., 2025). AI is a powerful tool to augment human intelligence, not replace breeders. For the foreseeable future, a breeder’s expertise, intuition, and creative vision will remain the soul of the improvement process (Moor et al., 2023). Overall, AI provides optimal mathematical solutions, while experienced breeders assess their real-world feasibility and suitability for local practices and markets.
